# The regulatory frameworks surrounding CRISPR‐edited papaya and their impact on international commerce

**DOI:** 10.1002/jsfa.70478

**Published:** 2026-01-26

**Authors:** Luíza Favaratto, Mirielson L da Silva, David S Buss, Oeber F Quadros, Raul Tapia‐Tussell, José A Ventura, Antonio Alberto R Fernandes, Patricia M B Fernandes

**Affiliations:** ^1^ Núcleo de Biotecnologia, Universidade Federal do Espírito Santo Vitória Brazil; ^2^ Unidad de Energía Renovable, Centro de Investigación Científica de Yucatán Merida México; ^3^ Instituto Capixaba de Pesquisa, Assistência Técnica e Extensão Rural Vitória Brazil; ^4^ Present address: Department of Agricultural and Resource Economics University of Saskatchewan, Saskatoon, Canada; ML Silva, Sante des Plants et Environnement, Institut Sophia Agrobiotech Antibes France

**Keywords:** exportation, gene editing, plant viruses, regulation

## Abstract

The papaya tree (*Carica papaya* L.), native to the Americas, is cultivated in tropical regions and holds substantial economic importance, with an estimated export volume of 365 000 t in 2023. However, diseases caused by viruses, fungi, bacteria, and nematodes can lead to severe losses. Among the more than 38 known viral diseases affecting papaya, only a few poses serious threats to cultivation, notably Papaya Ringspot, Papaya Mosaic, and Papaya Sticky Disease (PSD). Emerging technologies, particularly CRISPR/Cas9 gene editing, offer promising avenues to enhance plant resistance. This study examines regulatory paradigms in key papaya‐producing and importing countries, highlighting the need for international regulatory harmonization to reduce trade barriers and improve market access for CRISPR‐edited cultivars. We demonstrate the feasibility of CRISPR‐based genome editing in papaya (*Carica papaya* L.) by targeting phytoene desaturase as a proof‐of‐concept marker gene and *β‐*1,3‐glucanase, a resistance gene identified through proteomic profiling of host–pathogen interactions during infection by the papaya meleira virus (PMeV and PMeV2) complex. This virus complex causes PSD, a major threat to papaya production, rendering the fruit commercially unviable due to negative effects on texture and flavor as well as inhibiting the formation of benzyl isothiocyanate (BITC), and the fruits become susceptible to fruit flies, which are quarantine pests. Despite extensive traditional breeding efforts, resistant papaya genotypes have yet to be identified, underscoring the need for innovative approaches. However, translating advancements into commercial applications remains challenging due to the diverse and often inconsistent regulatory frameworks governing genome‐edited crops across different jurisdictions. © 2026 The Author(s). *Journal of the Science of Food and Agriculture* published by John Wiley & Sons Ltd on behalf of Society of Chemical Industry.

## INTRODUCTION

The export of papaya is of major economic importance to several tropical countries, with an estimated global export volume of 365 000 t in 2023.^1^ However, growers can suffer significant losses due to plant diseases, especially viruses such as Papaya Sticky Disease (PSD).^2^, ^3^

The global landscape of agriculture is undergoing a profound transformation with the advent of precision breeding technologies, particularly the CRISPR/Cas9 system (e.g., Pandey *et al*.^4^). This groundbreaking gene‐editing tool is fostering the development of crops with enhanced traits such as increased yield, improved nutritional content, and resistance to pests and diseases. CRISPR‐edited crops are becoming commercially available, for example, the GABA tomato, approved for sale in Japan, producing high amounts of γ‐aminobutyric acid (GABA), which gives health benefits such as reduced blood pressure.^5^ Furthermore, the successful editing of tomatoes to increase sweetness was recently published.^6^ In addition, the CRISPR/Cas9 system has been successfully used to enhance resistance against the papaya ringspot virus (PRSV) in melon (*Cucumis melo*).^7^ Those studies show the potential CRISPR technology has to enhance *Carica papaya* against viral diseases.

The transition of CRISPR‐edited crops from laboratory innovation to commercial deployment is hindered by divergent regulatory frameworks globally. A central challenge lies in the classification of this emerging technology relative to conventional methods of genetic modification. The term ‘Genetically Modified (GM) Organism’, established in the 1990s, lacks a universal definition. For instance, the Food and Agriculture Organization of the United Nations (FAO) defines GM organisms as ‘an organism such as a plant, animal or microorganism whose gene(s) have been altered using genetic modification techniques’.^8^ This broad characterization encompasses transgenic organisms and has historically triggered consumer skepticism and restrictive policies on GM cultivation and trade.

In contrast, Genetically Edited (GEd) organisms, developed through technologies such as CRISPR/Cas9, are increasingly framed as distinct from GM organisms. Proponents argue that gene editing enables precise, targeted and smaller modifications that if done without introducing foreign DNA, aligns outcomes with those achievable through traditional breeding. Regulatory distinctions often hinge on this premise. For example, the United States Department of Agriculture (USDA) exempts GEd crops from stringent oversight if the genetic alterations could theoretically arise naturally or through conventional breeding, provided no plant pathogens or exogenous genetic material are involved.^9^ This regulatory approach emphasizes product over process, diverging from the precautionary principles applied to transgenic organisms.^10^ Although, gene editing can be used to introduce exogenous DNA and, in that case, it would be considered and regulated as transgenic (GM).

**Figure 1:**
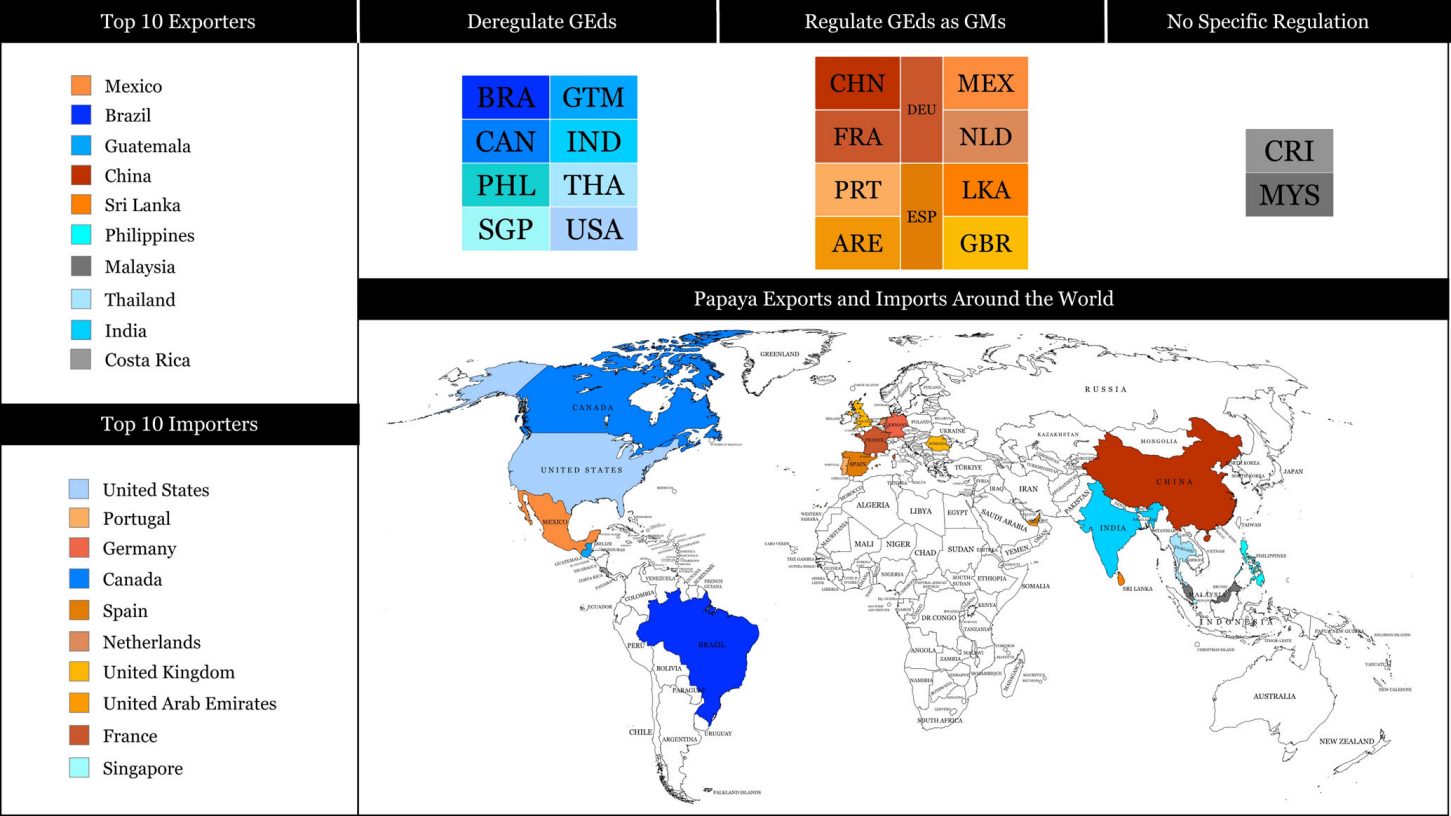
Top ten papaya exporting and importing (USD) countries and how they address GEd regulation. Data from FAOSTAT.^1^ The names of the countries are coded according to ISO 3166 alpha‐3 code. Map developed at https://www.mapchart.net/detworld.html.

The regulatory frameworks for GEd organisms, including CRISPR‐edited papaya, are not homogeneous and can differ significantly from those applied to GM organisms. In countries such as the United States and Brazil, CRISPR‐edited papaya without an exogenous gene will not be classified as GM, while conversely, in nations like Mexico, these edited plants will be subject to stringent GM regulations even without a foreign DNA. This regulatory diversity poses significant challenges for the international trade of CRISPR‐edited papaya. The ability of producers to navigate these regulatory landscapes effectively is crucial for the successful commercialization and global distribution of these crops (Fig. 1).

This study evaluates the potential of CRISPR/Cas9‐mediated genome editing to mitigate PSD by a case study targeting and modulating the expression of a key regulatory gene implicated in plant antiviral defense pathways. Furthermore, we aim to explore the regulatory frameworks governing CRISPR‐edited papaya in major producing and consuming countries, analyzing the implications of these regulations on international trade, and discussing the potential for harmonizing regulatory approaches to facilitate fair and efficient market access. By examining the example of CRISPR‐edited papaya, we seek to highlight the broader challenges and opportunities associated with the commercialization of GEd organisms in a globalized agricultural economy.

## WORLD TRADE IN PAPAYA

According to the FAO, global papaya production is dominated by India, the Dominican Republic, Mexico, Brazil, and Indonesia.^11^ However, a notable discrepancy exists between production volumes and export metrics, as these leading producers do not uniformly rank among the top five exporters in terms of quantity or economic value. While Mexico and Brazil maintain prominence in both production and export markets, other nations such as Guatemala demonstrate outsized contributions to export revenue despite lower production outputs. In terms of export volume, Brazil, Mexico, and Guatemala are accompanied by Malaysia and Sri Lanka as key contributors (Supporting Information, Table S1).

Although papaya is traded worldwide, exporters tend to focus on specific markets. The United States, as the leading importer, absorbs nearly the entire production of Mexican papayas^11^ and about 65% of Guatemalan output, with most of the remaining Guatemalan papayas going to El Salvador. In contrast, Brazilian papaya exports are more broadly distributed, with the European Union (EU) being the major importer.

Papaya import patterns also exhibit notable differences. The United States is the largest importer by both value and quantity, but the rankings diverge beyond the top spot. In terms of import value, members of the EU dominate, reflecting the higher prices paid in Europe. In contrast, the values for quantity are difficult to extract, as records are dominated by trade hubs, such as Singapore, the United Arab Emirates, and to an extent the Netherlands, act as both importers and exporters, with fruit passing through in transit.^12^ Despite having similar economic models, the regulatory landscape in each country varies considerably, as will be seen later. Whilst it would be helpful to have more precise import/output data from trade hubs this depends on the willingness of the countries concerned.

## REGULATION

Current regulatory frameworks predominantly prioritize oversight of imported and domestically marketed agricultural products, with minimal emphasis on export‐oriented commodities. While harmonization of phytosanitary and biotechnological regulations among major trading partners might be anticipated, significant discrepancies persist. A critical challenge lies in establishing clear definitions to distinguish GMs from GEds. Regulatory approaches to GEd crops can be categorized into three paradigms:

1. No regulatory distinction between GEd and GM:
  Malaysia, Mexico, United Arab Emirates, Guatemala, and El Salvador apply existing GM organism regulations to genome‐edited products, regardless of the absence of transgenes.^13^, ^14^, ^15^, ^16^
2. Explicit regulatory distinction between GEd and GM:
  Brazil, China, India, and the United States employ tiered frameworks that exempt certain GEd crops from strict GM requirements, contingent on the absence of foreign DNA.^17^, ^18^, ^19^, ^20^
3. Proposed distinction under deliberation:
  The EU (including member states such as the Netherlands) and Singapore are evaluating case‐specific risk assessments to potentially differentiate GEd crops from transgenic counterparts.^21^, ^22^

This regulatory fragmentation complicates international trade and stifles innovation in agricultural biotechnology, underscoring the urgent need for multilateral consensus on science‐based governance of genome‐edited crops.

### Export dynamics and regulatory landscapes in major papaya‐exporting nations

Despite its agricultural prominence, Mexico imposes stringent restrictions on both GM and GEd crops, permitting only commercial production of GM cotton.^23^ However, Mexico remains a major importer of GM crops, sourcing 50% of its cotton demand from the United States. It is also the world's second‐largest importer of GM corn and third‐largest importer of GM soybeans, predominantly from the United States and Brazil.^23^ This dichotomy reflects a reliance on imported biotechnology‐derived commodities despite domestic regulatory constraints.

As the second‐largest global producer of biotech crops,^24^ Brazil has approved 105 GM events, with adoption rates reaching 99% for soybeans and cotton, and 95% for corn.^25^ The National Technical Biosafety Commission (CTNBio) regulates GEd products under Normative Resolution 16 (NR‐16/2018), exempting non‐transgenic edits from stringent oversight.^26^ This framework facilitated Brazil's 2018 approval of its first CRISPR‐edited crop: high‐amylopectin corn.^19^ In 2022, CTNBio further classified CRISPR‐edited soybeans – developed by Embrapa to reduce anti‐nutritional factors – as conventional (non‐GM), exempting them from biotech regulations.^19^, ^25^ These policies place Brazil at the forefront of integrating advanced biotechnologies into agriculture.

Guatemala ranks third in papaya exports (Table 1), primarily supplying the United States (65%) and neighboring El Salvador with lower‐value fruit.^11^ As a member of the Central America Customs Union, Guatemala harmonizes biotech regulations with Honduras and El Salvador. While companies may apply for GM crop certification, no commercial GM or GEd crops are currently cultivated.^14^ Regulatory distinctions between GEd and GM crops remain ambiguous: legislation exempts organisms deemed obtainable through conventional breeding from ‘Living Modified Organism’ (LMO) classification. However, the LMO definition – any organism with a novel genetic combination via modern biotechnology – creates interpretive challenges.^27^

**Table 1:** The major exporting, importing, and producing countries for papaya according to the Food and Agriculture Organization of the United Nations^1^, ^11^ and World Integrated Trade Solution^12^

Country	Export		Import		Production (t)
	Value (1000 USD)	Quantity (t)	Value (1000 USD)	Quantity (t)	
MEX	122502	197928	—	—	1148545
BRA	53051	37864	—	—	1107761
GTM	21691	43426	14	21	85002
USA	20129	13719	165518	227319	4650
ESP	15504	7693	20807	9058	—
NLD	14242	3412	12179	4586	—
CHN	10661	7398	2597	2590	685320
EU	6252	3506	81299	31821	—
PRT	5828	2051	29097	12075	—
MYS	4691	14272	370	1057	38883
IND	3585	9484	—	—	5240000
DEU	2063	586	22937	7809	—
ARE	630	398	10720	4135	—
IDN	142	463	—	—	1238692
SGP	15	10	8610	15161	—
CAN	1	0.3	23815	18970	—
SLV	—	—	3217	14943	1535

USD (United States dollars). The names of the countries are coded according to ISO 3166 alpha‐3 code. IND: India, DOM: Dominican Republic, MEX: Mexico, BRA: Brazil, IDN: Indonesia, GTM: Guatemala, USA: United States of America, NLD: Netherlands, MYS: Malaysia, LKA: Sri Lank, PRT: Portugal, DEU: Germany, CAN: Canada, ESP: Spain, SGP: Singapore, ARE: United Arab Emirates, SLV: El Salvador.

These contrasting regulatory frameworks underscore the complexities of international agricultural trade. Mexico's reliance on biotech imports contrasts with Brazil's proactive adoption of genome editing, while Guatemala's ambiguous policies reflect broader regional uncertainties.

### Import dynamics and regulatory frameworks in key papaya‐importing markets

The United States is the global leader in GM crop cultivation, with 71.5 million hectares dedicated to GM soy, cotton, and corn in 2019, representing 38% of global biotech acreage.^28^ Regulatory oversight is managed by the Animal and Plant Health Inspection Service (APHIS/USDA), which exempts GEd crops lacking transgenes from stringent regulation, provided modifications align with natural genetic variation.^17^ The Food and Drug Administration (FDA) may intervene depending on trait‐specific risks. As the world's largest papaya importer (Table S1), the US is also one of only two nations (alongside China) commercializing GM papaya. Hawaiian production focuses almost exclusively on GM cultivars Rainbow and Sunup, though exports remain limited to Canada and Japan due to restrictive international GM policies.^29^

The EU, the second‐largest papaya importer, maintains rigorous restrictions on GM and GEd crop cultivation. Under the European Court of Justice 2018 ruling, CRISPR‐edited organisms are classified as GMs, subjecting them to the same regulatory hurdles as transgenic crops.^30^ Consequently, GM crop production is negligible, limited to approximately 47 500 ha of *Bacillus thuringiensis* (Bt) corn in Spain and Portugal.^30^ However, the EU is a major importer of GM commodities, sourcing > 30 million tonnes of GM soybeans and 20 million tonnes of GM corn annually, with adoption rates estimated at 90% (soy) and 20% (corn) in 2024.^30^ A 2023 European Commission proposal seeks to differentiate New Genomic Techniques (NGTs), NGT plants with edits indistinguishable from natural mutations (e.g., CRISPR base edits) would be exempt from GM regulations and NGT plants with non‐natural edits would follow existing GM protocols.^30^ This proposal led to 2 years of discussions within the EU regulatory system as reviewed in Council of the European Union Interinstitutional file 2023/0226(COD).^31^ A common position on NGTs has been agreed and at time of writing the next stage is presentation to the European Parliament. Under these proposals NGT Category 1 plants would be exempt from most EU GM regulations whilst Category 2 plants would continue to be regulated under current GM regulations. Category 1 NGT plants ‘are plants that could also occur naturally or be produced by conventional breeding techniques’ (EU_Interinstitutiona2023/0226(COD).^31^ The precise legal definition currently proposed (EU document 52023PC0411, Annex 1), includes for example, substitution or insertion of no more than 20 nucleotides, deletion of any number of nucleotides and targeted modification of any size, on the condition that the resulting DNA sequences already occur (possibly with insertion or deletion as described), in a species from the breeders' gene pool. Although CRISPR is not specifically mentioned, it is assumed that it would be defined as Category 1. The definition of NGT Category 1 is open to criticism (e.g., Vighi and De Storme^32^) and there are also numerous caveats, such as herbicide resistance cannot be included as an NGT Category 1, however, it is achieved.

#### Trade hubs: divergent regulatory approaches

In an era of globalization, trade hubs serve as critical nodes in the flow of agricultural commodities, yet their regulatory strategies for managing GM and GEd crops reveal stark contrasts. The Netherlands, as the EU's primary entry point for papaya, processes and re‐exports 74% of its imports under stringent EU GM regulations, while advocating for relaxed rules on NGTs to spur biotech advancement. Singapore, by contrast, imposes no oversight on transshipped GM/GEd papaya, deferring regulatory responsibility to destination countries, despite classifying such crops identically domestically. Meanwhile, the United Arab Emirates exemplifies a hybrid approach, aligning its transit regulations with importer requirements to expedite re‐exports.

Collectively, these case studies underscore a widening divide between proactive adopters of biotechnology, like the US, and cautious regulators, such as the EU. Trade hubs capitalize on these regulatory asymmetries to optimize commodity flows, yet the lack of harmonized definitions for GM and GEd crops, particularly under evolving policies like the EU's proposed NGT framework, risk fragmenting international standards. Balancing innovation, trade efficiency, and biosafety demands urgent collaboration to reconcile divergent paradigms and foster a coherent global approach.^11^, ^22^, ^33^, ^34^, ^35^

#### Bilateral agreements

Bilateral relations between agricultural producers and importers regarding biotechnology governance remain nuanced and dynamic, shaped by commodity‐specific economic imperatives and evolving regulatory harmonization efforts. A seminal example involves HB4 drought‐tolerant wheat, genetically modified to express a sunflower (*Helianthus annuus*) gene. In October 2020, Argentina conditionally approved its cultivation contingent on concurrent approval by Brazil, its primary export market. Brazil authorized milled HB4 wheat flour imports in November 2021 but restricted whole‐grain imports to prevent unintended cultivation.^25^, ^36^ Commercial cultivation within Brazil was later permitted, illustrating a phased bilateral alignment tailored to a high‐value commodity. However, such resource‐intensive negotiations may prove commercially prohibitive for lower‐total‐value crops like papaya, underscoring the need for multilateral frameworks.

A more scalable model emerged in 2023, when Brazil, Argentina, Paraguay, and Uruguay established the International Network for Biosafety of Modern Biotechnology Products to standardize risk‐assessment protocols and harmonize biotech regulations regionally. Similarly, Guatemala aligned its policies with key trading partners: harmonizing with El Salvador (a major papaya importer) in 2022 and Honduras in 2019 under the Central American Customs Union.^14^ These initiatives reflect growing recognition of regional regulatory cohesion as a precursor to efficient biotech trade, particularly for crops with transboundary value chains.

The global governance of gene‐edited crops is at a crossroads. The current patchwork of regulations, driven by a complex interplay of science, public perception, and powerful political economies, create inefficiencies and inequities that hinder agricultural innovation and trade. While regional harmonization is a positive step, it is insufficient. The urgent need is for a concerted multilateral effort to move beyond the entrenched GM organism debate. By implementing concrete mechanisms – such as a tiered regulatory model, mutual recognition of safety assessments, and a global transparency registry – the international community can foster a system that safeguards biosafety, facilitates trade, and unleashes the potential of biotechnology to address pressing global challenges in food security and sustainable agriculture. The choice is not between regulation and no regulation, but between fragmentation grounded in politicized caution and harmonization guided by scientific evidence and pragmatic cooperation.

## GENE EDITING IN PAPAYA – PROOF OF CONCEPT

**Figure 2:**
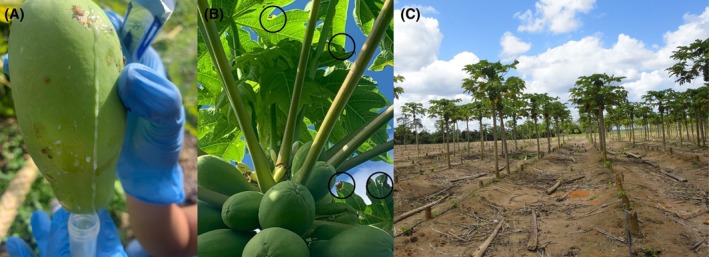
Symptoms of papaya sticky disease, leading to roguing. (A) Latex exudation from diseased fruit. (B) Necrotic lesions on leaves. (C) Roguing of diseased plants.

PSD, or meleira, was first confirmed to be a virus disease in Brazil in 1993^37^ and is already present in Mexico, Australia, and in Ecuador.^38^ PSD is characterized by the spontaneous exudation of fluid and latex from the fruit and tip burn on young leaves. Upon exposure to the atmosphere, the latex oxidizes, resulting in small necrotic lesions on young leaves and a sticky appearance of the fruit (Fig. 2(A),(B)), which renders them commercially unacceptable. Infected plants have reduced resistance to fruit flies, a quarantine pest for some countries, related to a lower presence of benzyl isothiocyanate (BITC), a natural chemical substance that has ovicidal action.^39^ Despite extensive efforts in Brazil and Mexico, identifying a papaya genotype resistant to PSD remains elusive.

Traditional plant breeding methods have proven ineffective, making roguing (Fig. 2(C)), by the systematic removal of infected plants, the primary control strategy. However, this method is compromised as symptoms only manifest after flowering, allowing infected but asymptomatic plants to go undetected for months, acting as hidden sources of inoculum.

The first successful CRISPR/Cas9 genome editing in papaya was reported in 2022, targeting the phytoene desaturase (*PDS*) gene to induce albino phenotypes by disrupting carotenoid pathway and impacting chlorophyll biosynthesis.^40^ Our study simultaneously explored the same locus using a different approach and also targeted the *β*‐1,3‐glucanase (*GLU*) resistance gene, selected through literature review and *Carica papaya* transcriptomic analysis.

Guide RNAs (gRNAs) were designed for target genes containing PAM (proto‐spacer adjacent motif) composed of an NGG sequence, necessary for the recognition of Cas9, and a restriction site (to *BssS∝1*) that functions as a mutation marker in the target, when digestion does not occur, indicating that the cleavage site has been lost due to mutation. The gRNAs were cloned into a modified binary vector (pKSE401)^41^ and transformed into *Agrobacterium tumefaciens* GV3101 for plant transformation using an adapted protocol.^42^ Two plant transformation strategies were used: (I) transient transformation in seedling leaves by vacuum agroinfiltration; (II) stable transformation in callus generated from hypocotyls – somatic embryos were regenerated on Murashige and Skoog medium supplemented with 2 mg L^−1^ each of benzylamino purine (BA) and naphthalene acetic acid (NAA) and rooted with 2 mg L^−1^ BA and 2 mg L^−1^ indole acetic acid (IAA) in activated charcoal medium (as described in Zhu *et al*.^43^).

As a result of the transient transformation, plants agroinfiltrated with the construct for the *PDS* target showed a chlorosis (yellowing) phenotype and foliar necrosis spots 7 days after inoculation (Supporting Information, Fig. S1), characteristics linked to the knockout of the phytoene desaturase gene. However, the phenotype of the mock plants (agroinfiltrated only with the plasmid without the construct) remained unchanged (Fig. S1(A)–(C)). The stable transformation was confirmed by molecular analysis (for both *PDS* and *GLU* genes) by polymerase chain reaction (PCR) and assays using restriction digestion. The fragments of PCRs that were not digested indicate the loss of the restriction site of the *BssS∝1* enzyme, confirming the mutation of the targets (Fig. S2).

To reinforce the effect of the *PDS* gene knockout, we isolated protoplasts from regenerated embryos, assessed viability by fluorescein diacetate staining (in fluorescence microscopy) (Fig. S3(A)), and analyzed chlorophyll autofluorescence by flow cytometry (CytoFLEX V2‐B2‐R0; Beckman Coulter, Inc., Brea, CA, USA), using cells whose pigments were removed with acetone as a control (Control SC). The protoplasts isolated from the *pds* knockout embryos showed lower fluorescence than those that did not receive the transformed plasmid (positive control for the presence of chlorophyll). In addition, *pds* knockout protoplasts showed similar results to those of cells whose pigments were removed (Control SC) (Fig. S3(B),(C)).

To assess the effect of the mutation in the *GLU* gene, we evaluated the callose deposition of cells cultured in suspension and stained with aniline blue (modified protocol from Herburger and Holzinger^44^) and visualized using a fluorescence microscope. As a result, *glu* knockout cells showed increased callose deposition when compared to the control group (agroinfiltrated with plasmid without the construct) (Fig. S4). Consistent results with findings that suppress this gene enhances callose accumulation and may restrict viral movement.^45^

These results align with prior CRISPR/Cas9 studies in papaya^40^ and other species such as *Arabidopsis thaliana* and *Medicago truncatula*,^46^ supporting the role of *β*‐1,3‐glucanase repression as a potential defense mechanism against viral spread. For more information on the experiments, please see Supporting Information Methos S1.

## THE FINANCIAL ASPECTS OF GENE EDITING IN PAPAYA

Global papaya trade dynamics, as summarized in Table S1, reveal that commercially available GM papaya cultivars currently exert minimal influence on international markets. The Rainbow cultivar, modified for resistance to PRSV, remains predominantly confined to the United States, Japan, and Canada due to restrictive GM policies in other regions. Similarly, China's Huanong GM papaya^47^ is primarily consumed domestically and though lacking comprehensive export data with 99% of its nominal exports disproportionately concentrated in Macau and Hong Kong, likely reflecting intraregional trade rather than global distribution.^12^

The expansion of GEd technologies has catalyzed global interest in developing GEd crops. According to the European Union's Scientific Advisory Group for Agricultural Innovation (EU‐SAGE) database, 854 publications on CRISPR‐edited crops were documented as of 2024, with research predominantly originating from China and the United States.^21^ Among these, only three studies focus on papaya, including investigations representing contributions from the United States, Thailand, and the United Kingdom.^40^, ^48^, ^49^ This surge in research reflects both the agronomic potential of GEd crops and anticipated reductions in regulatory barriers compared to transgenic counterparts, which carry significant economic implications. Regulatory classification as a GM organism escalates development‐to‐commercialization costs to approximately US$24.5 million per crop, whereas conventionally regulated crops incur costs of approximately US$10.5 million.^50^, ^51^ Conversely, restrictive policies, such as a hypothetical EU‐wide ban on GEd crops, could precipitate cumulative economic losses exceeding €1.2 trillion over 10 years due to diminished competitiveness.^50^ Despite regulatory challenges, commercialization of GEd crops is advancing: the first CRISPR‐edited mustard debuted in US markets in 2023, while the EU has initiated field trials of GEd rice in Italy, despite stringent regulations.^52^

The introduction of GEd papaya varieties presents a paradigmatic shift for global trade and production dynamics. Current legislative frameworks exhibit regulatory inertia, often lagging behind biotechnological innovation. Key questions persist: Will GEd papaya circumvent trade barriers imposed on transgenic crops? Can harmonized international standards mitigate market fragmentation? Addressing these uncertainties is critical, as divergent regulatory landscapes risk exacerbating trade asymmetries, particularly for developing economies reliant on papaya exports. Strategic alignment of policy with technological progress will be essential to harness the socioeconomic benefits of GEd papaya while ensuring equitable market access.

Papaya cultivars exhibit significant heterogeneity in market valuation, contingent upon geographical origin and perceived quality. For instance, Brazilian papaya commands a premium in the EU, achieving an average export price of US$1454 ton^−1^ in 2023, whereas Guatemalan exports to El Salvador averaged US$215 ton^−1^.^1^ The Mexico‐United States trade dyad represents the largest bilateral exchange, warranting specialized analysis. Mexico's comparatively stringent biosafety regulations contrast with the United State's permissive stance toward GEd crops, suggesting that Mexican adoption of GEd papaya varieties would face minimal market resistance from its primary importer. This inference is reinforced by Mexico's existing reliance on US‐sourced GM corn and soy, which dominate agricultural imports. However, this assessment overlooks domestic regulatory complexities and potential competition from Guatemala and Brazil. Brazil possesses the scientific infrastructure and progressive regulatory framework (e.g., CTNBio's case‐by‐case evaluations under NR‐16) to develop GEd papaya cultivars, potentially displacing Mexican exports in the US market. Such a shift, however, risks compromising Brazil's access to the lucrative EU market, where GEd crop regulations remain under review.

Trade dynamics are inherently shaped by intersecting political and economic imperatives. While geopolitical factors influence policy, the global trend increasingly favors deregulated trade for GEd crops, particularly techniques like CRISPR/Cas9. This trajectory is exemplified by multilateral agreements such as the 2023 International Network for Biosafety of Modern Biotechnology Products, which advocates harmonized risk‐assessment protocols. The integration of CRISPR‐edited papaya into global markets could significantly enhance yield stability and disease resistance, addressing critical constraints in tropical agriculture. However, divergent regulatory regimes such as the EU's precautionary approach *versus* the Americas’ innovation‐oriented policies threaten to fragment trade networks, disadvantaging producers in developing economies.

At the level of the producer, costs and benefits will depend on how access to the new genetic material is controlled (summarized recently in Pixley *et al*.^53^). Whilst improved crop yields and disease resistance will increase income and reduce uncertainty, there are concerns that the patenting of new crops will restrict farmer access and generate a monopoly. This is currently a major discussion point in the EU, with a proposal for a ban on patents, despite the risk this would pose to innovation.^54^ One possible solution would be the granting of licenses from the patent holder to multiple seed companies, which would then compete in the normal way, keeping grower costs down. Alternatively, licenses might be granted to not‐for‐profit organizations such as the African Agricultural Technology Foundation, allowing famer access to crops. A licensing system does need a testing and enforcement system to be effective. Nonetheless, the momentum around the world is to allow the use of new technology for plant improvement.

## CONCLUSIONS

CRISPR/Cas9 has emerged as a powerful tool for precisely, effectively, and sustainably enhancing plant resistance against devastating diseases that cause significant economic losses. The high specificity of this technique allows for the target modification of key genes, making it a more efficient alternative compared to traditional breeding methods, which have shown limited success and require extensive time to achieve the desired outcomes. The integration of CRISPR/Cas9 technology into the genetic improvement of *Carica papaya* marks a transformative advancement in agricultural biotechnology.

From a socioeconomic perspective, harmonizing international biosafety frameworks and preempting trade disputes are critical to prevent market fragmentation, particularly for developing economies reliant on papaya exports. Proactive alignment of policy frameworks with technological advancements is essential to realize the socioeconomic benefits of GEd papaya, ensuring equitable trade practices and fostering sustainable agricultural intensification. A potential model for this is the International Network for Biosafety of Modern Biotechnology Products between Brazil, Argentina, Paraguay, and Uruguay, standardizing risk‐assessment protocols and harmonizing biotech regulations regionally. Strategic collaboration between scientific, regulatory, and trade stakeholders will be pivotal in balancing innovation with biosafety, securing the long‐term viability of CRISPR‐edited crops in global markets.

## CONFLICT OF INTEREST

The authors have no relevant financial or non‐financial interests to disclose.

## ETHICS STATEMENT

This study complies with all ethical guidelines for biotechnology research established by the Brazilian National Technical Biosafety Commission (CTNBio) and the Internal Biosafety Commission (CIBio/UFES). No human or animal subjects were involved in this work. Funding sources are disclosed in the acknowledgments, and the authors declare no conflicts of interest. Data supporting this study is available upon reasonable request. Where applicable, permissions to reproduce third‐party materials have been obtained.

## AUTHOR CONTRIBUTIONS

OF Quadros, AAR Fernandes and PMB Fernandes conceived and designed the study. L Favaratto, ML Silva and OF Quadros conducted the experiments. All authors analyzed and discussed the results. L Favaratto, DS Buss, R Tapia‐Tussell, JA Ventura, AAR Fernandes and PMB Fernandes wrote the manuscript. All authors have read and agreed to the published version of the manuscript.

## Supporting information


**Figure S1.** Phenotypic evaluation of the transient effect of phytoene desaturase (*PDS*) gene knockout by CRISPR in *Carica papaya* seedling leaves 7 days after agroinfiltration with *Agrobacterium tumefaciens* GV3101. (A) Wild‐type leaves, mock condition (agroinfiltrated with empty plasmid). (B) Leaves agroinfiltrated with the CRISPR/Cas9 + gRNA transformation cassette for the target gene *PDS*. (C) Comparison of *Carica papaya* leaves in the mock condition (*) and in the *PDS* gene mutation condition (**). (D) The red arrow indicates the progression of the phytoene desaturase gene knockout effect at 1, 5, and 7 days after agroinfiltration, respectively. The red circles indicate tissue burn and necrosis due to the absence of chlorophyll for heat dissipation.

**Method S1.** Supplementary material.

**Figure S2.** Molecular analysis confirms the mutation of the *PDS* and *GLU* (*β*‐1,3‐glucanase) genes of *Carica papaya* by CRISPR/Cas9. Genomic DNA was extracted from agroinfiltrated callus and used for conventional PCR of the respective target genes; the PCR product was purified and used in a digestion assay. Lane M, Ladder Plus 1Kb marker. Lane 1, PCR fragments corresponding to 941 bp of the *GLU* target. Lane 2, BssS∝1 digestion of the amplicon presented in lane 1 (red arrow indicates the 941 bp fragment undigested by the enzyme due to the mutation). Lane 3, PCR fragments corresponding to 680 bp of the *PDS* target. Lane 4, BspH1 digestion of the amplicon presented in lane 3 (red arrow indicates the 680 bp fragment undigested by the enzyme due to the mutation).

**Figure S3.** Protoplasts of *Carica papaya pds* knockout show reduced chlorophyll content. (A) Isolated protoplasts (left) from transformed calluses were subjected to a fluorescence viability test with fluorescein diacetate (right). The culture was adjusted until 10^6^ cells mL^−1^ were acquired (20 000 events, 10 μL min^−1^). (B) Q2‐1 chlorophyll a fluorescence result in agroinfiltrated plants without the transformation vector (mock condition). (C) PD‐2 represents protoplasts isolated from embryos edited for *PDS* gene. A shift of the green peak to the right or left indicates increased or decreased chlorophyll autofluorescence, respectively. A sample treated with acetone to remove chlorophyll was used as a negative control (Control SC). Protoplasts were analysed using a CytoFLEX V2‐B2‐R0 cytometer (Beckman Coulter, Inc., Brea, CA, USA) equipped with violet (405 nm) and blue (488 nm) lasers and four fluorescence channels. Excitation occurred at 405 nm and chlorophyll autofluorescence was recorded in the 660/10 nm channel.

**Figure S4.** Cells of *Carica papaya glu* knockout by CRISPR/Cas9 show increased callose deposition. Aniline blue assay in cells edited for *β*‐1,3‐glucanase gene mutation. (A–F) and the control group – agroinfiltrated plants without the transformation vector (mock condition). (G–L) represents cells isolated from embryos edited for *GLU* gene. Panels A–C and G–I show cell clusters, while panels D–F and J–L depict single cells. A, D, G, and J are white light images. Assess callose deposition under UV light. Scale bar: 50 μm.

**Table S1.** Primer sequences, targeted gene and the restriction enzyme used in the CRISPR/Cas9 experiments.

## Data Availability

The data that support the findings of this study are available from the corresponding author upon reasonable request.
