# Supplementary figures and images for: The regulatory frameworks surrounding CRISPR‐edited papaya and their impact on international commerce
Source: J Sci Food Agric. 2026 Jan 26;106(11):6262–70. doi: 10.1002/jsfa.70478 (PMC13341055; doi:10.1002/jsfa.70478)

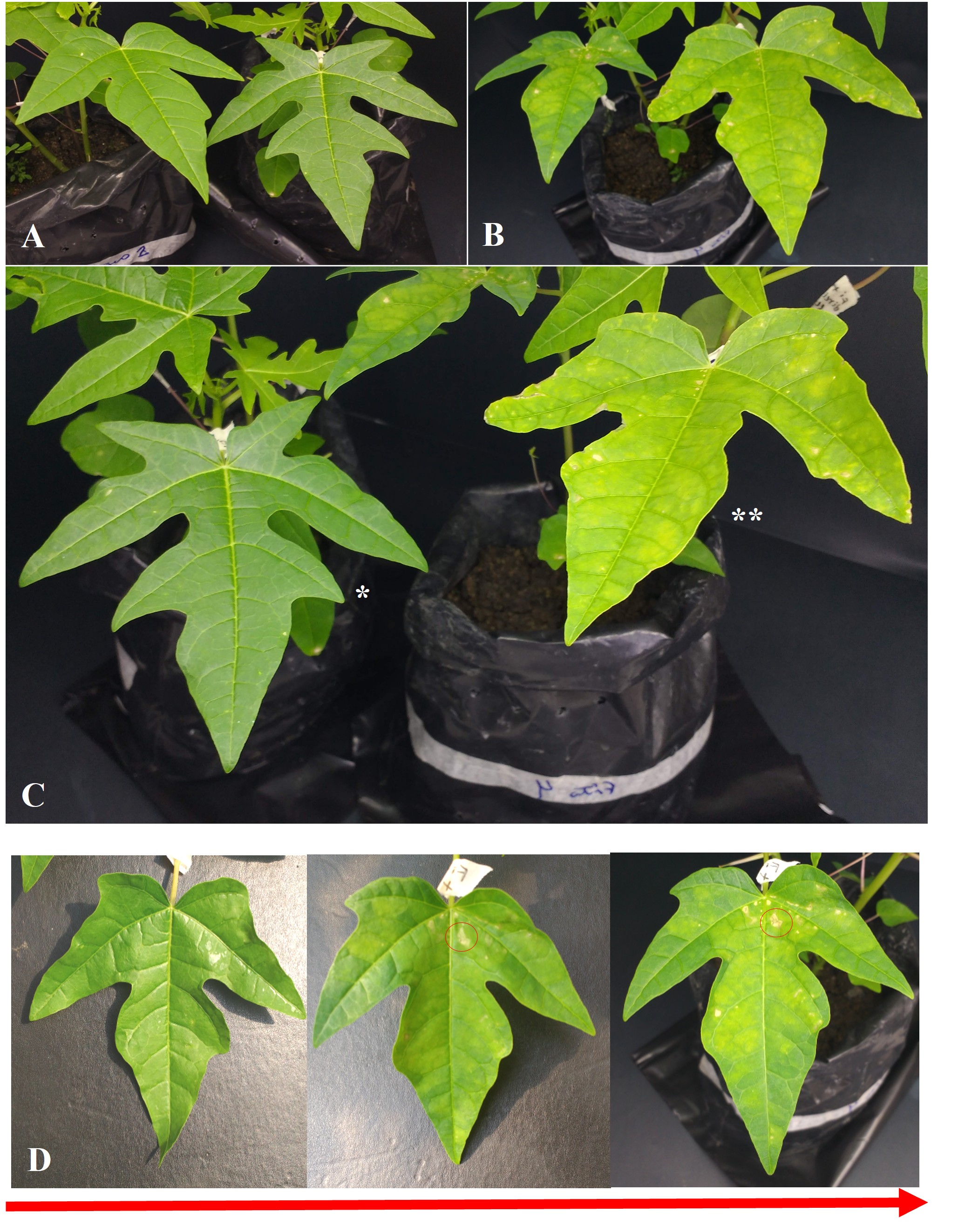

Supplement: Supplementary file 1 — Figure S1. Phenotypic evaluation of the transient effect of phytoene desaturase (PDS) gene knockout by CRISPR in Carica papaya seedling leaves 7 days after agroinfiltration with Agrobacterium tumefaciens GV3101. (A) Wild‐type leaves, mock condition (agroinfiltrated with empty plasmid). (B) Leaves agroinfiltrated with the CRISPR/Cas9 + gRNA transformation cassette for the target gene PDS. (C) Comparison of Carica papaya leaves in the mock condition (*) and in the PDS gene mutation condition (**). (D) The red arrow indicates the progression of the phytoene desaturase gene knockout effect at 1, 5, and 7 days after agroinfiltration, respectively. The red circles indicate tissue burn and necrosis due to the absence of chlorophyll for heat dissipation. [file JSFA-106-6262-s003.jpg]

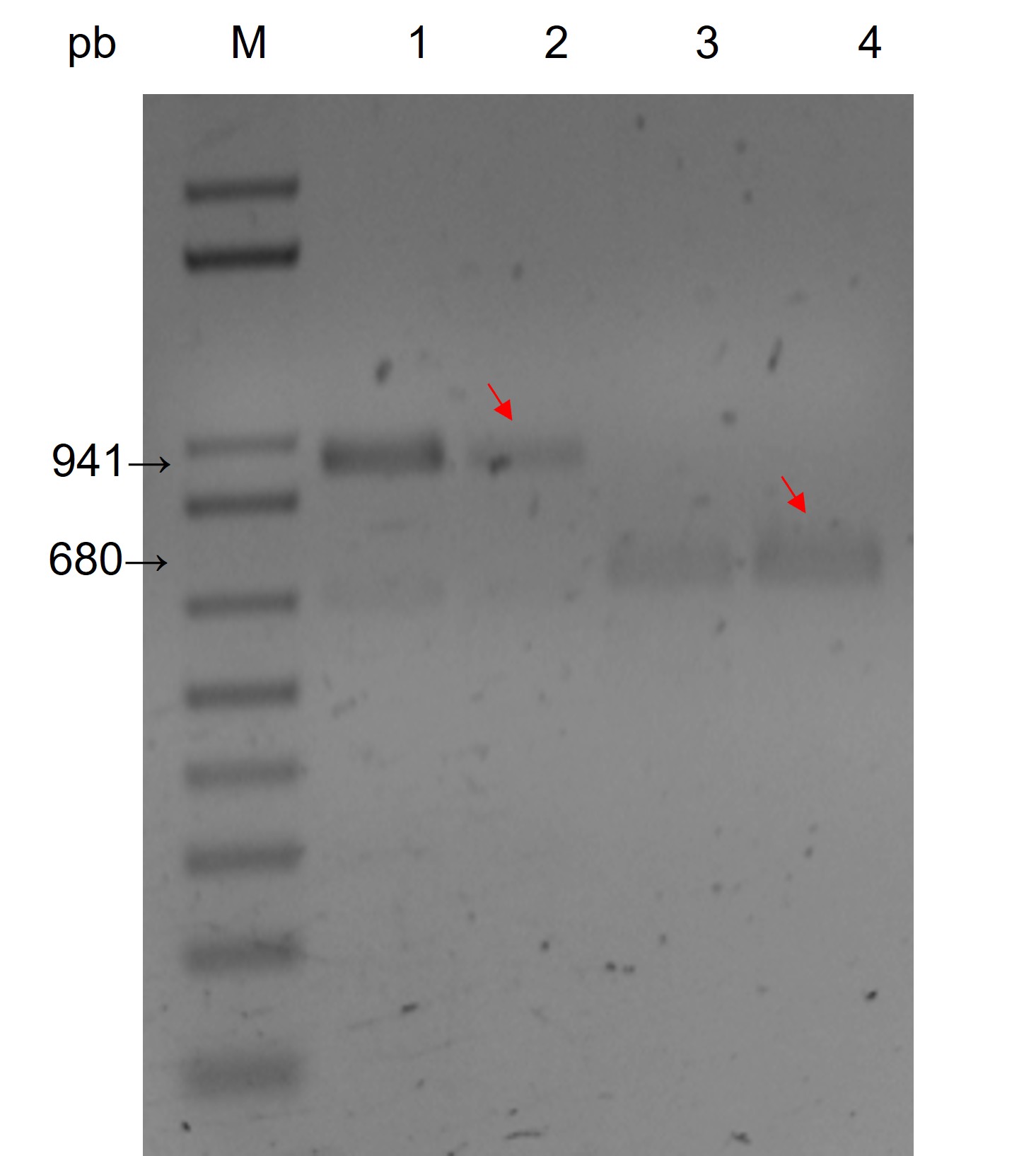

Supplement: Supplementary file 3 — Figure S2. Molecular analysis confirms the mutation of the PDS and GLU (β‐1,3‐glucanase) genes of Carica papaya by CRISPR/Cas9. Genomic DNA was extracted from agroinfiltrated callus and used for conventional PCR of the respective target genes; the PCR product was purified and used in a digestion assay. Lane M, Ladder Plus 1Kb marker. Lane 1, PCR fragments corresponding to 941 bp of the GLU target. Lane 2, BssS∝1 digestion of the amplicon presented in lane 1 (red arrow indicates the 941 bp fragment undigested by the enzyme due to the mutation). Lane 3, PCR fragments corresponding to 680 bp of the PDS target. Lane 4, BspH1 digestion of the amplicon presented in lane 3 (red arrow indicates the 680 bp fragment undigested by the enzyme due to the mutation). [file JSFA-106-6262-s006.jpg]

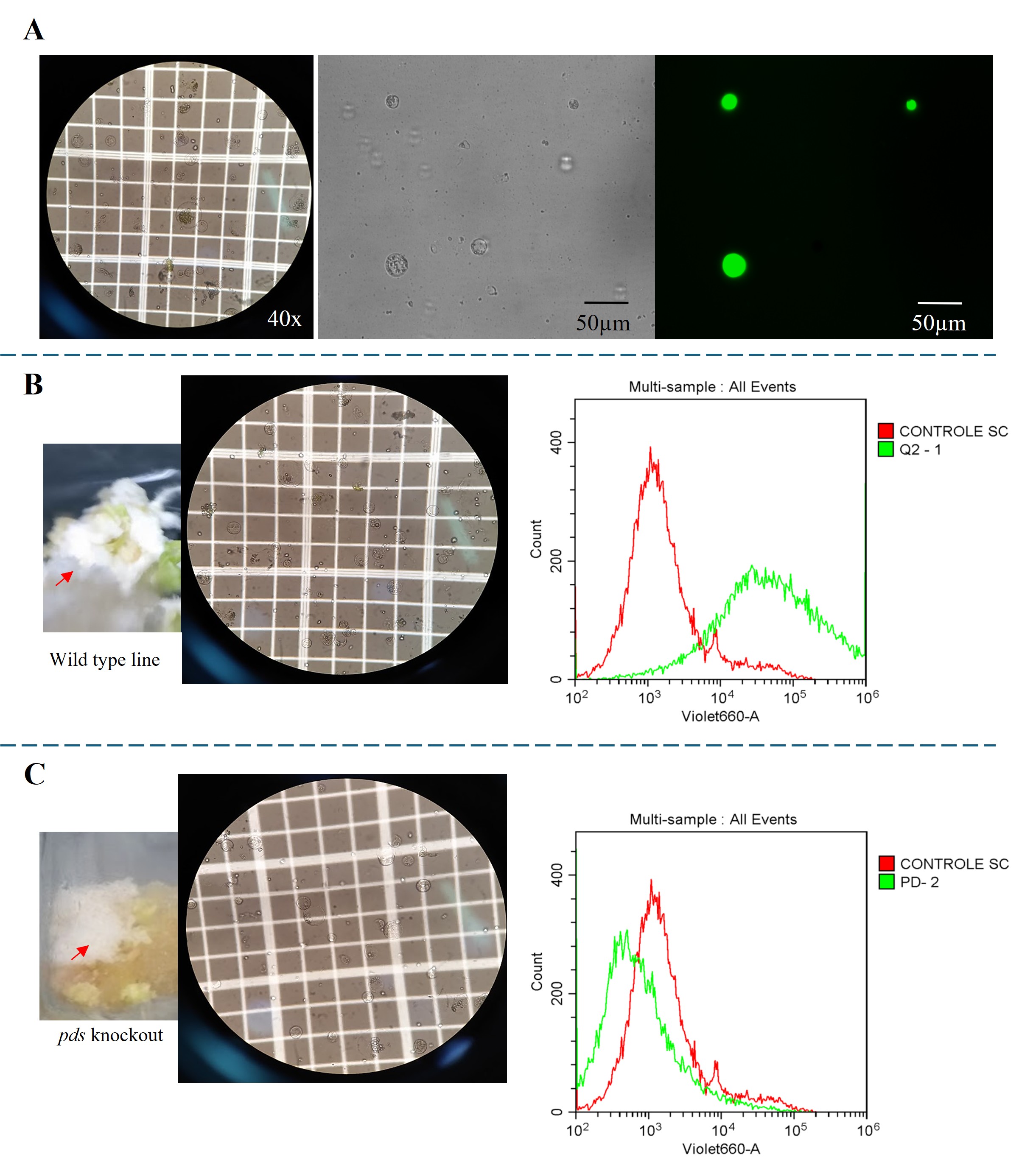

Supplement: Supplementary file 4 — Figure S3. Protoplasts of Carica papaya pds knockout show reduced chlorophyll content. (A) Isolated protoplasts (left) from transformed calluses were subjected to a fluorescence viability test with fluorescein diacetate (right). The culture was adjusted until 106 cells mL−1 were acquired (20 000 events, 10 μL min−1). (B) Q2‐1 chlorophyll a fluorescence result in agroinfiltrated plants without the transformation vector (mock condition). (C) PD‐2 represents protoplasts isolated from embryos edited for PDS gene. A shift of the green peak to the right or left indicates increased or decreased chlorophyll autofluorescence, respectively. A sample treated with acetone to remove chlorophyll was used as a negative control (Control SC). Protoplasts were analysed using a CytoFLEX V2‐B2‐R0 cytometer (Beckman Coulter, Inc., Brea, CA, USA) equipped with violet (405 nm) and blue (488 nm) lasers and four fluorescence channels. Excitation occurred at 405 nm and chlorophyll autofluorescence was recorded in the 660/10 nm channel. [file JSFA-106-6262-s004.jpg]

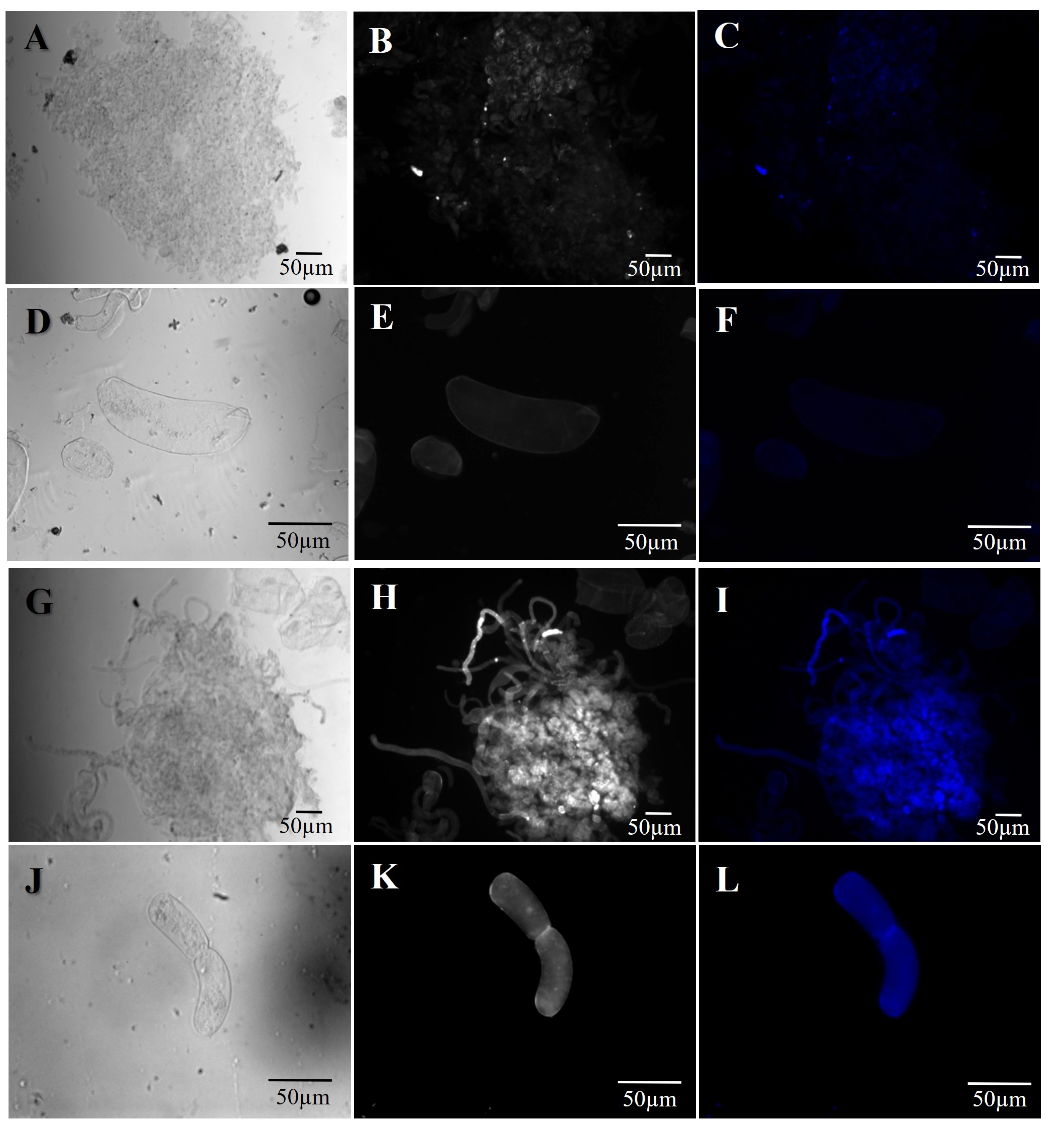

Supplement: Supplementary file 5 — Figure S4. Cells of Carica papaya glu knockout by CRISPR/Cas9 show increased callose deposition. Aniline blue assay in cells edited for β‐1,3‐glucanase gene mutation. (A–F) and the control group – agroinfiltrated plants without the transformation vector (mock condition). (G–L) represents cells isolated from embryos edited for GLU gene. Panels A–C and G–I show cell clusters, while panels D–F and J–L depict single cells. A, D, G, and J are white light images. Assess callose deposition under UV light. Scale bar: 50 μm. [file JSFA-106-6262-s001.jpg]
