# Supplementary material for: The regulatory frameworks surrounding CRISPR‐edited papaya and their impact on international commerce
Source: J Sci Food Agric. 2026 Jan 26;106(11):6262–70. doi: 10.1002/jsfa.70478 (PMC13341055; doi:10.1002/jsfa.70478)
Supplement: Supplementary file 2 — Method S1. Supplementary material. [file JSFA-106-6262-s002.docx]

**Supplementary material**

Papaya Sticky Disease (PSD), or “meleira,” is caused by two viruses: papaya meleira virus (PMeV) and papaya meleira virus 2 (PMeV2) (Sá Antunes et al., 2016; Maurastoni et al., 2023). The disease was confirmed to be caused by a virus in Brazil in 2003 (Maciel-Zambolim et al., 2003), followed by Mexico in 2008 (Perez-Brito et al., 2012). Australia reported PSD in 2014 (Pathania et al., 2019), and in 2021, papaya trees exhibiting PSD-like symptoms were observed in Ecuador (Quito-Avila et al., 2015). PSD is characterized by the spontaneous exudation of fluid and latex from the fruit and tip burn on young leaves. Upon exposure to the atmosphere, the latex oxidizes, resulting in small necrotic lesions on young leaves and a sticky appearance of the fruit (Fig. 2A-B).

This disease renders papaya fruits commercially unacceptable due to adverse effects on texture and flavor, effectively prohibiting exportation to international markets. Despite extensive efforts in Brazil and Mexico, identifying a papaya genotype resistant to PSD remains elusive.

Traditional plant breeding methods have proven ineffective, making roguing (Fig. 2C), or the systematic removal of infected plants, the primary control strategy. However, this method is compromised as symptoms only manifest after flowering, allowing infected but asymptomatic plants to go undetected for months, acting as hidden sources of inoculum.

The inaugural successful application of CRISPR-Cas9-mediated genome editing in papaya (*Carica papaya*) was documented in 2022, targeting the phytoene desaturase (*PDS*) gene to induce albino phenotypes through disruption of carotenoid biosynthesis (Brewer & Chambers, 2022). Independently, this study concurrently investigated the same genetic locus using a distinct methodological framework, while additionally prioritizing the β-1,3-glucanase resistance gene as a secondary target. Selection of these loci was informed by a systematic literature review and transcriptomic analyses conducted within the scope of this research. This gene was chosen as the target due to the action of β-1,3-glucanase on callose. The silencing of class I β-1,3-glucanases in transgenic tobacco mutants has been reported to reduce symptoms of tobacco mosaic virus (TMV) disease and to show increased callose deposition, thereby delaying traffic through plasmodesmata and hindering the dissemination of viruses and viral molecules (Bucher et al., 2001).

Previous studies conducted by our group showed that pre-fruiting papaya infected with the PMeV complex exhibit positive regulation of genes encoding callose synthesis and defense proteins and do not show visible symptoms of the disease. On the other hand, the analysis of symptomatic post-fruiting plants revealed a high presence of miRNAs that, according to the literature, are related to the post-transcriptional repression of genes encoding PR defense proteins, including β-1,3-glucanases. This analysis led us to presume that the repression of these transcripts is a defense mechanism to suppress β-1,3-glucanase synthesis, consequently leading to increased callose deposition in the plasmodesmata, characterizing a plant's attempt to hinder viral translocation through these channels. Calluses derived from CRISPR/Cas9 transfected cells showed increased cell wall callose deposition.

**METHODOLOGY OF GENE EDITING IN PAPAYA**

**Vector construction**

Guide RNAs (gRNAs) targeting the *Carica papaya* β-1,3-glucanase (*GLU*) and phytoene desaturase (*PDS*) genes were designed based on their coding sequences, ensuring target specificity by incorporating downstream PAM motifs and diagnostic restriction sites for mutational validation. The corresponding oligonucleotide pairs - *PDS*: *forward* 5′-ATTGATCTTAACAGCTCGTGCTT-3′, *reverse* 5′-AAACAAGCACGAGCTGTTAAGAT-3′; *GLU*: *forward* 5′-ATTGGATGCGATTCATGACTCTG-3′, *reverse* 5′-AAACCAGAGTCATGAATCGCATC-3′—were cloned into the CRISPR/Cas9 binary vector pKSE401 (Xing *et al.*, 2014) via Golden Gate assembly using *BsaI* and T4 DNA ligase in a single-tube reaction. Ligation products were electroporated into *Escherichia coli* DH5α, and transformants were selected on LB medium supplemented with kanamycin (50 µg/mL). Construction of the vectors was confirmed through plasmid DNA (extraction using ChargeSwitch™-Pro Plasmid Miniprep Kit - CS30250 InvitrogemTM) and PCR using cloning primers (Supplementary Table 1). The plasmid constructs called pKSPD (to PDS) and pKSBG (to GLU) were subsequently introduced into *Agrobacterium tumefaciens* GV3101 by electroporation, generating the strains for plant transformation assays.

**Papaya transformation and genotyping**

*Transient transformation*

*Agrobacterium* strain pKSPD was cultured in LB medium supplemented with kanamycin (50 µg·ml⁻¹) at 28 °C for 24 h; *A. tumefaciens* GV3101 lacking plasmid was grown in parallel without antibiotic as a negative control. Cultures were pelleted by centrifugation, washed twice with sterile distilled water and resuspended in half-strength Murashige and Skoog medium (½ MS; MS salts 2.16 g·L⁻¹, sucrose 30 g·L⁻¹, 2,4-D 2 mg·L⁻¹, pH 5.6–5.8) to an OD₆₀₀ of 0.5. Virulence was induced by addition of acetosyringone (20 µM) to the suspension followed by incubation at 28 °C for 3 h. For agroinfiltration, leaves were submerged in the bacterial suspension and subjected individually to vacuum infiltration (500 mmHg for 2 min) in a desiccator. Six plants were infiltrated in total (four with pKSPD and two with plasmid-free GV3101 as negative controls) and maintained in a greenhouse at ambient temperature and photoperiod; phenotypes were monitored photographically for 7 days post-infiltration.

*Stable transformation*

Plant tissue culture and *Agrobacterium tumefaciens*-mediated transformation were performed using a modified protocol adapted from Carlos-Hilario and Christopher (2015). Seeds of papaya (*Carica papaya* cv. THB) were surface-sterilized and germinated aseptically on 1% (w/v) agar medium. Hypocotyl and leaf explants were excised from two-week-old seedlings and cultured on Callus Induction Medium (CIM) for 30 days under controlled photoperiod conditions (16 h light/8 h dark) Resulting calluses were transferred to Liquid Callus Induction Medium (LCIM) to establish suspension cultures, which were subsequently co-cultivated with *A. tumefaciens* harboring the CRISPR-Cas9 construct (pKSPD and pKSBG).

Post-transformation, calluses were transferred to hormone-free CIM solid medium for one month to promote somatic embryogenesis. Mature embryos were then subcultured onto Murashige and Skoog (MS) basal medium supplemented with 1.0 mg L⁻¹ benzylaminopurine (BAP) and 0.5 mg L⁻¹ naphthalene acetic acid (NAA) to induce shoot and leaf organogenesis. Regenerated plantlets with fully expanded leaves were transferred to rooting medium containing 0.5 mg L⁻¹ indole-3-butyric acid (IBA) and 0.1 mg L⁻¹ indole-3-acetic acid (IAA), amended with 0.3% (w/v) activated charcoal to mitigate phenolic exudation (Zhu et al., 2006; Carlos-Hilario and Christopher, 2015).

*DNA extraction and molecular analysis*

The stable transformation was confirmed by molecular analysis. Genomic DNA was extracted from callus tissue (100 mg). Tissues were homogenized in DNA extraction buffer (500 mM NaCl, 100 mM Tris-HCl pH 7.5, 50 mM EDTA pH 7.5), followed by lysis with SDS (20%) at 65 °C. Lysates were purified through sequential phenol:chloroform:isoamyl alcohol (25:24:1) extractions, and DNA was precipitated with isopropanol, washed with 70% ethanol, and resuspended in RNase-treated Milli-Q water. DNA quality and concentration were determined by spectrophotometry (NanoDrop 2000, Thermo Scientific).

Genomic regions spanning the CRISPR target sites were amplified using gene-specific primers (Supplementary Table 1). PCR conditions consisted of an initial denaturation at 94 °C for 3 min, followed by 35 cycles of 94 °C for 45 s, 53 °C for 30 s, and 72 °C for 30 s, with a final extension at 72 °C for 3 min. Amplicons were digested with the corresponding restriction enzymes (*BssS∝1* for *PDS* and *BspH1* for *GLU* target) and resolved on 1% agarose gels in 1× TAE buffer. Gels were stained with ethidium bromide (1 mg/mL), and fragment sizes were estimated against a 1 kb DNA ladder (Invitrogen). Images were captured using an L-PIX digital gel documentation system (Loccus Biotecnologia).

**Effectiveness of gene editing**

*Protoplast isolation*

Protoplasts were isolated from CRISPR-edited somatic embryos and from pKSPD-transformed papaya suspension cultures following Zhang et al. (2011), with modifications. Embryogenic tissues were incubated in an enzymatic solution containing 1.2% (w/v) cellulase R-10, 0.3% (w/v) macerozyme R-10, 0.52 M D-mannitol, 20 mM KCl, and 20 mM MES. The solution was preheated to 55 °C for 10 min to reduce protease activity and improve enzyme solubility, cooled to room temperature, and supplemented with 10 mM CaCl₂ and 0.5% (w/v) PVP, followed by 0.22-µm sterile filtration. Tissues were digested for 13 h at 26 °C in the dark with gentle agitation (60 rpm).

*Protoplast purification and quantification*

Digested material was diluted in W5 buffer (154 mM NaCl, 125 mM CaCl₂, 5 mM KCl, 2 mM MES, 5 mM D-glucose), filtered through a 70-µm nylon mesh, and centrifuged at 800 rpm for 5 min. Cells were washed twice in W5 and resuspended in the same buffer. Protoplast concentration was determined using a hemocytometer after a 5-min settling period, with yields expressed as protoplasts g⁻¹ fresh weight (n ≥ 3).

*Viability analysis*

Viability was assessed using fluorescein diacetate (FDA). Suspensions were incubated with 12 µL FDA stock (5 mg/mL in acetone) per 500 µL of cells. Fluorescence was visualized using a Nikon Ti-Eclipse inverted microscope equipped with a 365/12-nm excitation and 397-nm emission filter set. Images were acquired with NIS-Elements AR 4.20, and viability was calculated as the percentage of FDA-positive cells relative to total visible protoplasts.

*Flow cytometry for mutation validation*

Protoplasts from CRISPR-edited somatic embryos were analyzed using a CytoFLEX V2-B2-R0 flow cytometer (Beckman Coulter) featuring 405-nm (80 mW) and 488-nm (50 mW) lasers. Samples were excited at 405 nm, and chlorophyll autofluorescence was detected through a 660/10-nm filter. 20,000 events were acquired per sample at 10 µL/min. Acetone-treated protoplasts served as non-fluorescent controls. Sample concentrations were maintained below 10⁶ cells/mL.

*Callose fluorescence assay*

One week after Agrobacterium-mediated transformation with strain pKSBG, suspension cells were evaluated for callose deposition following Herburger & Holzinger (2016). Cells were washed in LCIM medium and incubated in 8 N NaOH at 60 °C for 20 min. After centrifugation (1000 × g), samples were washed three times with Milli-Q water. A Sørensen phosphate buffer (0.1 M, pH 8.0) was prepared from 0.2 M NaH₂PO₄ and Na₂HPO₄ stock solutions and supplemented with 1% (w/v) aniline blue immediately before staining. Cells were incubated in staining solution for 1 h at room temperature in the dark with gentle agitation. Excess stain was removed with three washes in Sørensen buffer.

Fluorescence was visualized using a Nikon Ti-Eclipse microscope with UV excitation (390-nm bandpass) and emission (460-nm bandpass) filters. Images were collected and processed in NIS-Elements AR 4.20.

**Supplementary Figure legends**

**Supplementary Figure 1.** Phenotypic evaluation of the transient effect of phytoene desaturase (PDS) gene knockout by CRISPR in *C. papaya* seedling leaves 7 days after agroinfiltration with *A. thumefaciens* GV3101. A) Wild-type leaves, mock condition (agroinfiltrated with empty plasmid). B) Leaves agroinfiltrated with the CRISPR/Cas9 + gRNA transformation cassette for the target gene PSD. C) Comparison of *C. papaya* leaves in the mock condition (*) and in the PDS gene mutation condition (**). D) The red arrow indicates the progression of the phytoene desaturase gene knockout effect at 1, 5, and 7 days after agroinfiltration, respectively. The red circles indicate tissue burn and necrosis due to the absence of chlorophyll for heat dissipation.

**Supplementary Figure 2.** Molecular analysis confirms the mutation of the PDS and GLU (β-1,3-glucanase) genes of *C. papaya* by CRISPR/Cas9. Genomic DNA was extracted from agroinfiltrated callus and used for conventional PCR of the respective target genes; the PCR product was purified and used in a digestion assay. M) Ladder Plus 1Kb marker. 1) PCR fragments corresponding to 941 bp of the GLU target. 2) BssS∝1 digestion of the amplicon presented in 1 (red arrow indicates the 941 bp fragment undigested by the enzyme due to the mutation). 3) PCR fragments corresponding to 680 bp of the PDS targe. 4) BspH1 digestion of the amplicon presented in 3 (red arrow indicates the 680 bp fragment undigested by the enzyme due to the mutation).

**Supplementary Figure 3.** Protoplasts of *C. papaya* pds knockout show reduced chlorophyll content. A) Isolated protoplasts (left) from transformed calluses were subjected to a fluorescence viability test with FDA (right). The culture was adjusted until 10⁶ cells/mL were acquired (20,000 events, 10 µL/min). B) Q2-1 chlorophyll a fluorescence result in agroinfiltrated plants without the transformation vector (mock condition). C) PD-2 represents protoplasts isolated from embryos edited for PDS gene. A shift of the green peak to the right or left indicates increased or decreased chlorophyll autofluorescence, respectively. A sample treated with acetone to remove chlorophyll was used as a negative control (Control SC). Protoplasts were analyzed using a CytoFLEX V2-B2-R0 cytometer (Beckman Coulter, Inc., Brea, CA) equipped with violet (405 nm) and blue (488 nm) lasers and four fluorescence channels. Excitation occurred at 405 nm and chlorophyll autofluorescence was recorded in the 660/10 nm channel.

**Supplementary Figure 4.** Cells of *C. papaya* glu knockout by CRISPR/Cas9 show increased callose deposition. Aniline blue assay in cells edited for β-1,3-glucanase gene mutation. (A-F) and the control group - agroinfiltrated plants without the transformation vector (mock condition). (G-L) represents cells isolated from embryos edited for GLU gene. Panels A-C and G-I show cell clusters, while panels D-F and J-L depict single cells. A, D, G, and J are white light images. Assess callose deposition under UV light. Scale bar: 50 μm.
