# Supplementary material for: The regulatory frameworks surrounding CRISPR‐edited papaya and their impact on international commerce
Source: J Sci Food Agric. 2026 Jan 26;106(11):6262–70. doi: 10.1002/jsfa.70478 (PMC13341055; doi:10.1002/jsfa.70478)
Supplement: Supplementary file 6 — Table S1. Primer sequences, targeted gene and the restriction enzyme used in the CRISPR/Cas9 experiments. [file JSFA-106-6262-s005.docx]

| **Primer name** | **Primer sequence** | **Target** | **Amplicon size** | **Restriction enzyme** |
| --- | --- | --- | --- | --- |
| Phydes Fw | 5’-ATTGATCTTAACAGCTCGTGCTT-3’ | Phytoene desaturase cloning | 423 bp | *BssS∝1* |
| Phydes Rv | 5’-AAACAAGCACGAGCTGTTAAGAT-3 |  |  |  |
| Glucan Fw | 5’-GGATGCGATTCATGACTCTG-3’ | β-1,3-glucanase cloning | 423 bp | *BspH1* |
| Glucan Rv | 5’-AAACCAGAGTCATGAATCGCATC-3 |  |  |  |
| Primer 181 Fw | 5’-ATGACTTTATGCGGGAGTGT-3’ | Phytoene desaturase diagnosis | 680 bp | *BssS∝1* |
| Primer 500 Rv | 5’-GACGTAATGATTGAGTTAGAA-3’ |  |  |  |
| Primer 941 Fw | 5’-CTGCTCAGATAGGTGTTTGC-3’ | β-1,3-glucanase diagnosis | 941 bp | *BspH1* |
| Primer 941 Rv | 5’-TCAGTTGAAACTGACTGGAT-3’ |  |  |  |

**Supplementary Table 1.** Primer sequences, targeted gene and the restriction ezyme used in the CRISPR-Cas9 experiments.
